# OLC1在肺鳞癌组织中的过表达与患者的不良预后相关

**DOI:** 10.3779/j.issn.1009-3419.2017.05.08

**Published:** 2017-05-20

**Authors:** 坤鹏 张, 格非 赵, 汀 肖, 萍 李, 杰 宋, 燕宁 高, 克林 孙

**Affiliations:** 100021 北京，国家癌症中心/中国医学科学院北京协和医学院肿瘤医院，分子肿瘤学国家重点实验室癌发生与预防分子机理北京市重点实验室 State Key Laboratory of Molecular Oncology, Beijing Key Laboratory for Carcinogenesis and Cancer Prevention, National Cancer Center/Cancer Hospital, Chinese Academy of Medical Sciences and Peking Union Medical College, Beijing 100021, China

**Keywords:** OLC1, 免疫组织化学, 肺鳞癌, 预后, OLC1, Immunohistochemical staining, Lung squamous cell carcinoma, Prognosis

## Abstract

**背景与目的:**

OLC1（overexpressed in lung cancer 1）是本实验室前期工作中筛选克隆出的一个新型肺癌相关基因。它在肺癌和其它恶性肿瘤中均有较高表达，并与食管鳞癌、卵巢癌、乳腺癌、结直肠癌患者的不良预后相关。本研究旨在检测OLC1在肺鳞癌（squamous cell carcinoma, SCC）和腺癌（adenocarcinoma, ADC）患者肿瘤组织中的表达情况，研究其与肺癌患者预后之间的关系。

**方法:**

分别对108例肺鳞癌和90例肺腺癌的癌组织进行免疫组织化学染色，检测OLC1蛋白的表达水平，分析OLC1的表达水平与临床特征及预后的关系。

**结果:**

OLC1在肺腺癌组织中的过表达率显著高于鳞癌（87.5% *vs* 55.3%, *P* < 0.001）。OLC1在癌组织中的过表达与肺腺癌患者的预后无显著相关性，与肺鳞癌患者的预后关系的单因素分析显示两者存在相关性（*P*=0.042），而多因素分析则显示OLC1过表达与鳞癌患者预后的相关性无统计学意义（*P*=0.05）。

**结论:**

OLC1在肺腺癌组织中的过表达高于鳞癌，在癌组织中的过表达仅与鳞癌患者的不良预后相关，但不能作为影响预后的独立危险因素。

## Abstract (en)

**Background and objective:**

*OLC1* (overexpressed in lung cancer 1), screened out and cloned in our previous research, is a new gene associated with lung cancer. It is highly expressed in lung cancer and many other malignant tumors, and is associated with poor prognosis of esophageal squamous cell carcinoma, ovarian cancer, breast cancer and colorectal cancer. The aim of this research was to detect the expression level of OLC1 in the tumor tissues of lung adenocarcinoma (ADC) and squamous cell carcinoma (SCC) and explore its relationship with the prognosis of lung cancer patients.

**Methods:**

Lung cancer tissues of 108 SCC and 90 ADC was dealed with immunohistochemical staining to detect the expression level of OLC1. The relationship between the expression level of OLC1 and clinical parameters and prognosis was analyzed.

**Results:**

The rate of high expression of OLC1 staining in ADC was significantly higher than that in SCC (87.5% *vs* 55.3%, *P* < 0.001). The overexpression of OLC1 in tumor tissues did not have a significant relationship with the prognosis of patients with ADC, but it was related with a poor prognosis of SCC patients as the univariate analysis showed. However the multivariate regression analysis showed that correlation between the overexpression of OLC1 and poor prognosis of SCC patients did not have a statistical significance (*P*=0.05).

**Conclusion:**

The expression of OLC1 in ADC might be higher than that in SCC. A higher score of OLC1 staining in tumor tissue was associated with a poorer prognosis of patients with SCC, but could not be an independent predictor for a shorter overall survival in patients with SCC.

肺癌是世界上最常见的恶性肿瘤之一，发病率及死亡率均居各种恶性肿瘤首位，发病率呈逐年上升趋势^[1]^。目前已知多种基因在肺癌发生发展过程中起到重要作用^[2]^。随着分子生物学和蛋白质组学等技术的发展，寻找肺癌发生、发展相关的基因，研究它们的表达与肺癌的诊断、预后判断和治疗的关系，一直是基础和临床共同努力的目标^[3]^。

为了进一步研究肺癌的分子机制，本实验室前期工作中通过抑制性消减杂交文库（suppression subtractive hybridization, SSH）等高通量手段建立了中国人肺癌相关差异表达基因文库^[4]^。通过功能筛选，成功克隆出在肺癌组织中表达显著升高的肺癌相关基因*OLC1*（overexpressed in lung cancer 1），并通过免疫组化等证实OLC1在肺腺癌、鳞癌、小细胞肺癌组织中均有较高阳性表达率^[5]^。在肺癌患者外周血中的OLC1蛋白水平，也显著高于正常健康对照组^[6]^。有研究报道，OLC1在食管鳞癌、卵巢癌、乳腺癌、结直肠癌组织中均检测到较高阳性表达，并与不良预后相关^[7-10]^。但OLC1在肺癌患者组织中的表达与预后的关系尚不确切，因此本研究的目的在于揭示OLC1在肺癌组织中的表达与临床特征和预后之间的关系。

## 1 材料和方法

### 1.1 材料

肺癌组织芯片：上海芯超生物科技有限公司（HLug-Ade180Sur-01, HLug-Squ150Sur-01），两张组织芯片分别包含90例肺腺癌和75例肺鳞癌患者的癌组织，年龄30岁-84岁，平均年龄62.3岁。所有患者均接受外科手术切除肿瘤组织，手术时间2004年7月-2009年6月，所有患者均具有完整的术前临床信息、术后病理分型分期及随访信息，随访截至2014年8月。另外33例鳞癌标本来源于2008年1月-2009年4月在中国医学科学院肿瘤医院接受外科手术切除的肺癌患者，取自病理科石蜡切片。年龄41岁-74岁，平均年龄58.3岁，患者具有完整临床及随访信息，随访截至2015年11月。

### 1.2 方法

组织芯片和石蜡切片的的免疫组化均采用SP法。免疫组化一抗来源于实验室前期制备，利用OLC1全长蛋白作为抗原制作的单克隆鼠抗，通过Western blot检测筛选后获得^[6]^。一抗工作浓度1:100，1×PBS稀释，室温孵育1 h。二抗来源于购自北京中杉金桥公司的即用型山羊抗鼠/兔通用型IgG，二抗室温孵育20 min，DAB显色，在显微镜下控制染色强度，苏木素染核1 min-2 min，乙醇逐步脱水后树胶封片。本研究采用1×PBS代替一抗作为阴性对照，采用过表达的肺癌组织切片作为阳性对照。

### 1.3 结果判定

染色结果由两位病理医师独立阅片，判断结果。根据切片在显微镜下观察，由细胞着色强度评分和阳性细胞百分比评分之积进行判定。阳性细胞百分比评分：阳性率≤5%计0分，5% < 阳性率≤25%计1分，25% < 阳性率≤50%计2分，50% < 阳性率≤75%计3分，75% < 阳性率≤100%计4分。着色强度评分：无色0分，淡黄色1分，棕黄色2分，棕褐色3分。取两者乘积进行分级：0分为（-），1-4分为（+），5-8分为（++），9-12分为（+++）。本研究将（+++）定义为OLC1蛋白过表达。

### 1.4 统计学处理

结果采用SPSS 19.0进行统计分析，OLC1在肺癌组织中的过表达与临床特征之间的关系中分类变量采用卡方检验。与预后之间的关系采用*Kaplan-Meier*进行单因素分析，再利用*Cox*多因素回归分析验证各影响因素与总体生存期的关系，*P* < 0.05为差异有统计学意义。

## 2 结果

### 2.1 OLC1在肺癌组织中的表达情况

在198例组织中，组织芯片免疫组化染色脱片7例，最终纳入研究191例，其中腺癌88例，鳞癌103例，总体5年生存率为44.4%，鳞癌5年生存率为56.5%，腺癌患者5年生存率为30%。免疫组化染色的结果显示OLC1在非小细胞肺癌组织中表达存在差异（图 1），OLC1在88例肺腺癌中的过表达率为87.5%，103例肺鳞癌中的过表达率为55.3%，差异有统计学意义（*P* < 0.001）。经卡方检验分析，OLC1在腺癌组织中的过表达与年龄、性别、病理分级、肿瘤直径、分期和淋巴结转移均无明显相关性。在103例肺鳞癌患者中，OLC1过表达与临床特征的分析显示，OLC1在年龄≥60岁（*P*=0.002）的患者中更易出现过表达，OLC1的过表达与肿瘤分化程度、性别、肿瘤分期、是否淋巴结转移、肿瘤直径无关（表 1）。

**1:**
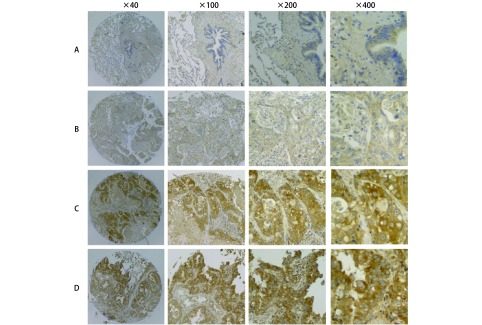
OLC1在不同组织中的表达情况。利用OLC1单克隆鼠抗体在组织芯片中用免疫组织化学染色检测OLC1在癌旁正常肺组织和肺癌组织中的表达。A：OLC1在正常肺组织中不表达；B：OLC1在鳞癌中弱表达；C：OLC1在鳞癌中高表达；D：OLC1在腺癌中高表达。 The expression of OLC1 in different tissues OLC1 expression in adjacent normal lung tissues and lung cancer tissues, as observed by immunohistochemical staining with a rat monoclonal antibody against OLC1 applied to the tissue microarrays. A: OLC1 was not expressed in adjacent normal lung tissues; B: OLC1 was lowly expressed in squamous cell carcinoma tissues; C: OLC1 was overexpressed in squamous cell carcinoma tissues; D: OLC1 was overexpressed in lung adenocarcinoma tissues.

**1:** OLC1在鳞癌组织中的表达与临床特征的关系 Association between clinical features and OLC1 expression in Lung SCC tissues

Clinical parameters	Total (*n*=103)	Intensity		*P*
		-/+/++ (*n*=46)	+++ (*n*=57)	
Age (yr)				0.002
< 60	37	24 (64.9%)	13 (35.1%)	
≥60	66	22 (33.3%)	44 (66.7%)	
Gender				0.264
Male	97	42 (43.3%)	55 (56.7%)	
Female	6	4 (66.7%)	2 (33.3%)	
Pathological grade				0.076
High/Middle	78	31 (39.7%)	47 (60.3%)	
Low	25	15 (60.0%)	10 (40.0%)	
Lymph node metastasis^*^				0.411
No	55	23 (41.8%)	32 (58.2%)	
Yes	46	23 (50.0%)	23 (50.0%)	
Clinical stage^*^				0.855
Ⅰ/Ⅱ	69	31 (44.9%)	38 (55.1%)	
Ⅲ/Ⅳ	32	15 (46.9%)	17 (53.1%)	
^*^: 2 of them lymph node and clinical stage uncertain.				

### 2.2 OLC1在肺腺癌和鳞癌组织中的过表达与预后的关系

对肺腺癌患者进行的*Kaplan-Meier*生存分析显示，OLC1的过表达与肺腺癌患者的预后无关（*P*=0.816）。肺鳞癌患者的生存资料单因素分析显示OLC1在癌组织中的过表达与不良预后存在相关性（*Log-rank* *χ*^2^=4.12，*P*=0.042，图 2A），OLC1过表达组与非过表达组的5年生存率分别为50.9%和67.4%。为了排除混杂因素影响，我们采用*Cox*多因素回归分析进行验证。纳入与预后关系较为密切的性别、年龄、分化程度、TNM分期以及OLC1在癌组织中过表达，共5个因素进行*Cox*多因素回归分析。结果显示，OLC1表达不同的两组之间总生存差异无统计学意义（*P*=0.05，表 2，图 2B）。OLC1在癌组织中的表达与年龄存在一定的关联，在≥60岁的患者中更易表达，以上的分析显示OLC1过表达和年龄≥60都是可能影响预后的重要危险因素，本研究将OLC1过表达和年龄两个因素联合进行分析。单因素*Kaplan-Meier*生存分析显示，OLC1过表达且年龄≥60岁组的预后显著差于OLC1非过表达且年龄 < 60岁组（*log-rank* *χ*^2^=8.392，*P*=0.004，图 3A）。纳入性别、分化程度、TNM分期共同进行多因素分析也显示，鳞癌患者OLC1过表达且年龄≥60岁时，预后显著变差（HR=5.125，95%CI 1.756-14.96，*P*=0.003，图 3B）。

**2:**
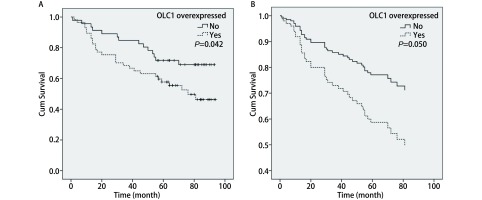
OLC1在鳞癌组织中的过表达与预后的关系。A：过表达组与非过表达组*Kaplan-Meier*生存曲线，*Log-rank*检验提示OLC1高表达组比低表达组预后差，差异有统计学意义（*P* =0.042, *n* =103）。B：多因素*Cox*回归分析显示OLC1过表达组的预后较差，但差异无统计学意义（*P* =0.05, *n* =103）。 Correlation of the OLC1 overexpression in lung SCC tissues with prognosis. A:*Kaplan-Meier* survival curve of patients with or without OLC1 overexpression in lung SCC tissues, *Log-rank* test showed that the OLC1 overexpressed group had a poorer prognosis (*P* =0.042, *n* =103). B: Multivariate *Cox* regression analysis showed that the OLC1 overexpressed group had a poorer prognosis than another group, without statistical significance (*P* =0.046, *n* =65).

**2:** 肺鳞癌患者总生存期的单因素和*Cox*多因素分析 The univariate and multivariate *Cox* analysis for overall survival in lung SCC patients

Variable	Univariable (*n*=103)		Multivariable Model 1 (*n*=103)				Multivariable Model 2 (*n*=103)		
	*P*		HR	95%CI	*P*		HR	95%CI	*P*
Gender									
Male/Female	0.242		4.401	1.367-14.174	0.013		4.304	1.331-13.913	0.015
Age (yr)									
< 60/≥60	0.005		2.207	0.980-4.968	0.056		-	-	-
Grade of differentiation									
High-middle/Low	0.028		1.211	0.563-2.605	0.624		1.225	0.569-2.635	0.604
Clinical stage									
Ⅰ, Ⅱ/Ⅲ, Ⅴ	0.002		2.903	1.347-6.259	0.007		2.912	1.354-6.260	0.006
OLC1 overexpression									
No/Yes	0.042		2.048	1.000-4.194	0.050		-	-	-
Age & OLC1 overexpression	0.013								0.007
^#^/≥60 or OLC1 overexpressed	0.062		-	-	-		2.817	0.922-8.601	0.069
^#^/≥60 & OLC1 overexpressed	0.004		-	-	-		5.125	1.756-14.983	0.003
^#^: < 60 & OLC1 not overexpressed.									

**3:**
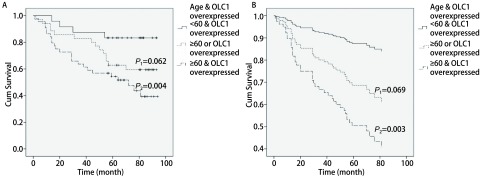
OLC1过表达与年龄≥60岁共同影响鳞癌患者的预后A：单因素*Kaplan-Meier*生存分析显示，OLC1过表达且年龄≥60岁组比OLC1非过表达且年龄 < 60岁组生存显著降低（*P*_2_=0.004）、仅OLC1过表达或年龄≥60岁不能作为影响鳞癌患者预后的独立危险因素（*P*_1_=0.062）；B：多因素*Cox*回归分析显示，OLC1过表达且年龄≥60岁组的生存较差（*P*_2_=0.003）。 OLC1 overexpressed combined with age ≥60 years together influence the prognosis of lung SCC patients. A: Univariate *Kaplan-Meier* survival curve showed that the OLC1 overexpressed & age ≥60 years group had a poorer prognosis (*P*_2_=0.004). Only with one of the factors of OLC1 overexpressed and age ≥60 years could not be an independent risk factor influence the prognosis of lung SCC (*P*_1_=0.062). B: Multivariate *Cox* regression analysis showed that the OLC1 overexpressed & age≥60 years group had a poorer prognosis (*P*_2_=0.003)

## 3 讨论

肺癌作为世界上发病率和死亡率均居各种恶性肿瘤首位的疾病^[1]^，在肺癌的发生发展过程中有一系列的基因和蛋白发生差异性表达，参与调节细胞周期和凋亡等步骤。关于肺癌发病的分子病理机制以及基因和蛋白的功能、表达水平改变的研究一直是寻找肺癌治疗新方法的重要途径。OLC1是在肺鳞癌新鲜肿瘤组织中筛选克隆出的肺癌相关新基因，定位于16q22.2^[4]^。在本课题组前期研究中，OLC1在肺癌患者外周血浆中的蛋白水平也显著高于健康人。OLC1的瞬时表达能引起IkappaB-α的磷酸化，使NF-kappaB向细胞核移位，从而激活NF-kappaB通路。在肺鳞癌的发生早期即不典型增生和原位癌阶段，就已经可以检测到OLC1表达的升高，因此推测OLC1可能通过激活NF-kapppaB通路介导肺癌早期癌变的发生^[5]^。

OLC1的表达可能与肺癌患者的吸烟史以及鳞癌的进展存在一定关联。张晓静等^[11]^通过制备香烟凝集物作用于肺癌细胞系的研究表明，香烟凝集物通过抑制OLC1蛋白泛素化降解过程，引起OLC1的高表达，OLC1的高表达可能通过促进BRCA1蛋白泛素化降解下调，导致抑癌基因BRCA1表达下调。有吸烟史的鳞癌患者外周血浆中OLC1蛋白水平明显高于无吸烟史患者^[6]^，并与鳞癌患者的吸烟史直接相关，可能提示吸烟致癌的原理和OLC1与鳞癌发生发展的重要关系。而本研究结果显示，OLC1尽管在腺癌组织中的表达率明显高于鳞癌，但在鳞癌中的差异表达可能提示OLC1与鳞癌的预后及临床特点关系更为密切。而由于本研究为回顾性研究，在组织芯片的临床信息中未包含准确的吸烟史信息，未能对OLC1在组织中的表达与吸烟的关系进行分析。

既往研究及本研究结果提示OLC1可能与多种肿瘤患者的预后相关。在关于OLC1在食管鳞癌中的研究表明，OLC1高表达以后，可能通过阻碍天冬氨酸特异性半胱氨酸蛋白酶-3（caspase-3）的激活和增强B淋巴细胞瘤-2（Bcl-2）的稳定性来抑制细胞凋亡，从而引起恶性肿瘤的发生和进展^[9]^。OLC1不仅在肺癌组织中高表达，研究表明，OLC1还在乳腺癌、卵巢癌、食管鳞癌、结肠癌组织中高度表达，并与不良预后直接相关^[7-10]^。因此OLC1可能是具有原癌基因属性的，并且它的表达调控有可能参与多种恶性肿瘤的发生和发展过程，但关于OLC1如何发挥调节功能，参与这些肿瘤进展的机制尚缺乏进一步研究。在乳腺癌、卵巢癌、结肠癌研究中的结果显示，OLC1在癌组织中表达阳性（++/+++）是与术后的不良预后直接相关的，在卵巢癌组织中OLC1高、低表达组的5年生存率分别为24.8%和75.2%^[8]^。本研究对OLC1的表达与肺鳞癌患者预后进行生存分析，我们首先按照OLC1表达阴性（-/+）和阳性（++/+++）进行分组，两组之间的生存无统计学差异（*P*=0.153）。进一步按照是否为强阳性表达进行分组发现，OLC1在鳞癌组织中的过表达（+++）与不良预后相关，过表达组与非过表达组5年生存率分别为50.9%和67.4%，单因素分析显示两组间预后存在显著差异（*P*=0.042），提示在本研究中OLC1的表达与肺鳞癌患者的不良预后存在一定的关系：鳞癌组织中OLC1表达较强可能提示患者手术治疗后预后更差。进一步纳入性别、年龄、分化程度、TNM分期以及OLC1在癌组织中过表达这5个影响因素，进行多因素*Cox*回归分析发现，OLC1差异表达的两组之间生存期无统计差异（*P*=0.05），仅有TNM分期可以作为影响患者预后的独立危险因素。但可以看到多因素回归分析中，OLC1过表达的P值处于临界状态，而本研究为单中心回顾性小样本手术治疗患者资料，可能存在偏倚，因此并不能完全否定OLC1过表达预测鳞癌患者预后的价值，未来仍需扩大样本量、同时考虑辅助治疗等因素的作用，进一步研究OLC1过表达对鳞癌患者预后的影响。

OLC1的过表达与年龄相关，并可能与年龄一起共同影响鳞癌患者的预后。OLC1在鳞癌患者中的过表达与临床因素进行分析后的结果分析显示，OLC1在年龄≥60岁的患者中更易表达。对鳞癌患者的预后资料进行的多因素分析发现，OLC1在癌组织中的过表达和年龄≥60岁是除TNM分期以外可能影响鳞癌患者预后的重要因素。因此本研究对OLC1过表达和年龄两个因素联合后进行分析，结果显示OLC1非过表达且年龄 < 60岁患者具有最好的预后，OLC1过表达且年龄≥60岁组的预后显著变差。说明OLC1的过表达可能与年龄≥60岁相关并共同影响鳞癌患者的预后，可能作为鳞癌患者预后的预测因素。

综上所述，OLC1在腺癌中的过表达率明显高于鳞癌，但目前研究并未发现OLC1在癌组织中的过表达与腺癌的预后相关。OLC1在鳞癌组织中的过表达与年龄呈相关性，更易在≥60岁的鳞癌患者组织中表达，且OLC1在鳞癌组织中的过表达与患者的不良预后存在一定的关联，因此OLC1在鳞癌组织中表达的研究可能为发现新的肺鳞癌患者预后预测因素，进行危险分层指导综合治疗提供帮助。未来仍需进一步研究OLC1表达差异的原因和影响预后的机制。
